# Supplementary material for: Porcupine: A visual pipeline tool for neuroimaging analysis
Source: PLoS Comput Biol. 2018 May 10;14(5):e1006064. doi: 10.1371/journal.pcbi.1006064 (PMC5963801; doi:10.1371/journal.pcbi.1006064)
Supplement: S1 File — (PDF) [file pcbi.1006064.s001.pdf]

Supporting information

S1 Docker files Porcupine provides a Docker image that creates the necessary run time environment for a pipeline that is constructed in the workflow editor. As with all generated code, the Docker code is fully customisable to a researcher's need, but our suggested scaffold requires only a single manual edit to be built as a Docker image. A Docker script can only refer to online or on-disk resources, so the pipeline file needs to be saved manually and added to the Docker file:

```
ADD /path/to/pipeline/script.py /somewhere/porcupipeline.py
CMD ["python", "/somewhere/porcupipeline.py"]
```

Once this line is added, the docker image can be built:

```
$ docker build -t mydockerimage -f Dockerfile
```

The output from the executed pipeline can be written to a local directory on a researcher's computer ('/my/local/directory') by mounting it to the docker output directory ('/data') with the '-v' option and running the image as if it were a standalone application.

```
$ docker run -v /my/local/directory:/data mydockerimage
```

This Docker command will execute the pipeline: load the data from an online repository, process the data, and store only the output data to a local directory. A fully worked out example with detailed explanation can be found here.
